# Pituitary adenylate cyclase-activating polypeptide prevents contrast-induced nephropathy in a novel mouse model

**DOI:** 10.1002/phy2.163

**Published:** 2013-11-19

**Authors:** Altaf-M Khan, Jerome L Maderdrut, Min Li, Herman L Toliver, David H Coy, Eric E Simon, Vecihi Batuman

**Affiliations:** 1 Department of Medicine, Section of Nephrology and Hypertension, Tulane University School of MedicineNew Orleans, Louisiana; 2 Department of Medicine, Peptide Research Laboratory, Tulane University School of MedicineNew Orleans, Louisiana; 3 Department of Veterans Affairs, Southeast Louisiana Veterans Health Care SystemNew Orleans, Louisiana

**Keywords:** Acute kidney injury, apoptosis, innate immunity, kidney injury biomarkers, renoprotection

## Abstract

We determined whether pituitary adenylate cyclase-activating polypeptide 38 (PACAP38) prevents contrast-induced nephropathy using human renal proximal tubule epithelial (HK-2) cells and homozygous endothelial nitric oxide synthase-deficient (eNOS^−/−^) mice as a novel in vivo model. Cultured HK-2 cells were pretreated with 10^−9^–10^−6^ mol/L PACAP or vasoactive intestinal peptide (VIP) for 1 h, and then exposed to ionic (Urografin) or nonionic (iohexol) contrast media at 50 mg iodine/mL for 24 h. Male eNOS^−/−^ mice received Urografin (1.85 g iodine/kg) intravenously after water deprivation for 24 h, and PACAP38 (10 *μ*g) intraperitoneally 1 h before and 12 h after Urografin injection. Urografin and iohexol increased lactate dehydrogenase and kidney injury molecule 1 in the culture medium, induced apoptosis, and inhibited cell proliferation in HK-2 cell cultures. PACAP38 and VIP reduced these changes in a dose-dependent manner. PACAP38 was more potent than VIP. In eNOS^−/−^ mice, Urografin raised serum creatinine and cystatin C levels, caused renal tubule damage, induced apoptosis, and promoted neutrophil influx. Urografin also increased kidney protein levels of proinflammatory cytokines, and kidney mRNA levels of proinflammatory cytokines, kidney injury biomarkers, and enzymes responsible for reactive oxygen and nitrogen species. PACAP38 significantly reduced these Urografin-induced changes in eNOS^−/−^ mice. This study shows that both Urografin and iohexol are toxic to HK-2 cells, but Urografin is more toxic than iohexol. Urografin causes acute kidney injury in eNOS^−/−^ mice. PACAP38 protects HK-2 cells and mouse kidneys from contrast media and is a potential therapeutic agent for contrast-induced nephropathy.

## Introduction

Iodinated radiocontrast media are used for a wide range of diagnostic and interventional procedures. They are usually classified as ionic or nonionic. Iodinated radiocontrast media are organic compounds containing covalently bound iodine and are either monomeric or dimeric molecules with three or six atoms of iodine, respectively (Thomsen and Morcos 2000). Iodinated radiocontrast media are relatively safe drugs, but can cause serious anaphylactic reactions or contrast-induced nephropathy in some patients. The incidence of contrast-induced nephropathy in the general clinical population is low (1–2%), but can be above 50% in vulnerable clinical subpopulations, such as elderly diabetic patients with chronic kidney disease (McCullough 2008; Thomsen et al. 2008). Nonionic iodinated radiocontrast media are less nephrotoxic than ionic iodinated radiocontrast media. Contrast-induced nephropathy is the third leading cause of iatrogenic acute kidney injury (Nash et al. 2002). The risk factors for development of contrast-induced nephropathy include preexisting kidney disease, diabetes mellitus, hypertension, cardiovascular disease, age, sickle cell anemia, gout flares, multiple myeloma, hypoalbuminemia, concurrent administration of nephrotoxic drugs, and dehydration (McCullough et al. 2006; McCullough 2008). The risk factors are additive, with preexisting kidney disease being the most potent risk factor. Contrast media damage the kidney due to a direct toxic effect of iodine on renal epithelial and endothelial cells, stimulation of innate immune responses and oxidative stress in the kidney, and reduction in renal blood flow (McCullough 2008; Sendeski 2011).

Pituitary adenylate cyclase-activating polypeptide (PACAP) was isolated from the hypothalamus in our laboratories during a screen for novel hypophysiotropic factors (Miyata et al. 1989, 1990), but it soon became clear that it is a pleiotropic peptide with potent anti-inflammatory and cytoprotective properties (Ganea and Delgado 2002; Vaudry et al. 2009). The cytoprotective effects of PACAP have been extensively studied in the brain and kidney. PACAP exists as two *α*-amidated peptides with 38 (PACAP38) or 27 (PACAP27) amino acids. It is a member of the secretin/growth hormone-releasing hormone (GHRH)/vasoactive intestinal peptide (VIP) family, and PACAP27 has 68% sequence identity with VIP. PACAP is most abundant in the brain, but there are significant levels in other organs, including the thymus, spleen, lymph nodes, and duodenal mucosa (Vaudry et al. 2009). It binds to three Class B (Class II) G-protein-coupled glycoprotein receptors that are coupled to multiple signal transduction pathways (Segre and Goldring 1993; Harmar et al. 2012). The “second” messengers include adenylate cyclase, phospholipase C, mitogen-activated protein (MAP) kinases, and calcium. PACAP binds to the PAC_1_ receptor with a high affinity, but it also binds to the VIP1 (VPAC_1_) and VIP2 (VPAC_2_) receptors with affinities comparable to VIP. In contrast, VIP binds to the PAC_1_ receptor with an affinity about 1,000 times less than PACAP (Vaudry et al. 2009; Harmar et al. 2012).

Pituitary adenylate cyclase-activating polypeptide has previously been shown to protect the kidney against injury due to ischemia/reperfusion (I/R) (Riera et al. 2001; Li et al. 2010; Khan et al. 2012), immunoglobulin light chain overload (Arimura et al. 2006), and a wide variety of nephrotoxins, including gentamicin, cisplatin, cyclosporine A, tacrolimus (FK506), and methotrexate (Li et al. 2008, 2011; Coy et al. 2011; Khan et al. 2011). We have, therefore, tested PACAP38 as a renoprotectant against contrast-induced nephropathy using a novel preclinical mouse model.

## Material and Methods

### HK-2 cell culture

HK-2 human renal proximal tubule epithelial cells were purchased from American Type Culture Collection (Manassas, VA) and were maintained in Gibco Keratinocyte Serum-Free medium supplemented with 5 ng/mL of recombinant epidermal growth factor and 0.05 mg/mL of bovine pituitary extract (Invitrogen, Carlsbad, CA). These cells were routinely cultured at 37°C in a humidified incubator with 95% air–5% CO_2_ and medium was replaced every 3–4 days. The cells were reseeded into 6-well or 96-well plates with the desired level of confluence (80%) for the various types of experiments.

### HK-2 cell apoptosis and cytotoxicity assays

To determine the optimal dose of iodinated radiocontrast media for induction of apoptosis, HK-2 cells, in supplement free medium, were exposed to either iohexol or Urografin (sodium diatrizoate and meglumine diatrizoate) at concentrations of 25–100 mg I/mL for 24 h and cell apoptosis was measured with an enzyme-linked immunosorbent assay (ELISA) for determination of mono- and oligonucleosomes using a Cell Death Detection ELISA^Plus^ kit (Roche Applied Science, Eugene, OR). To determine the dose–response curves for inhibition of contrast-induced apoptosis, HK-2 cells were treated with 10^−9^–10^−6^ mol/L PACAP or VIP for 1 h prior to exposing them to Urografin at 50 mg I/mL for 24 h. The effects of 10^−9^–10^−6^ mol/L PACAP38 or VIP on the release of the cytoplasmic enzyme lactate dehydrogenase (LDH) into the HK-2 cell culture medium after 24 h of exposure to Urografin (50 mg I/mL) were determined with an LDH activity assay kit (Sigma, St. Louis, MO) according to the manufacturer's instructions.

### HK-2 cell proliferation and kidney injury biomarker assays

The effects of various concentrations PACAP38 or VIP (10^−9^–10^−6^ mol/L) on the contrast-induced inhibition of HK-2 cell proliferation were assessed by determining the conversion of 3-(4,5-dimethylthiazol-2-yl)-2,5-diphenyltetrazolium bromide (MTT) to a purple formazan by reductases in intact cells (Promega, Madison, WI). One hour after treating the cell cultures with PACAP38 or VIP, confluent HK-2 cells were exposed to either iohexol or Urografin (50 mg I/mL) for 24 h. The effect of PACAP38 (10^−6^ mol/L) on the release of the kidney injury biomarker (Ichimura et al. 1998) kidney injury molecule 1 (KIM-1) into the HK-2 cell culture medium caused by 24 h of exposure to either iohexol or Urografin (50 mg I/mL) was determined with an ELISA kit (R&D Systems, Minneapolis, MN).

### Animal experiments

All animal experiments were performed under a protocol approved by the Institutional Animal Care and Use Committee at Tulane University. Male homozygous endothelial nitric oxide synthase (eNOS)-deficient mice (8–10 weeks old, *n* = 8) were obtained from Jackson Laboratory (Bar Harbor, ME). For the contrast medium-treated group, mice were water deprived for 24 h and then given an intravenous injection of Urografin (1.85 g iodine/kg). The contrast medium-treated group received PACAP38 (10 *μ*g/100 *μ*L) intraperitoneally 1 h before and 12 h after the injection of Urografin. Control mice received an equal volume of saline on the same schedule. All mice were euthanized at 72 h; blood and kidneys were collected for analyses.

### Quantification of functional and morphological kidney injury

To estimate renal function, we measured both creatinine and cystatin C in mouse serum. Serum creatinine was measured by isotope dilution liquid chromatography mass spectrometry/mass spectrometry at the UAB-UCSD O'Brien Core Center (Birmingham, AL). Tandem mass spectrometry was used to determine creatinine levels because the standard colorimetric method usually overestimates creatinine levels in mouse serum due to endogenous chromogens (Meyer et al. 1985). Serum cystatin C was measured with an ELISA kit (R&D Systems). For histological and immunohistochemical studies, formalin-fixed 4-*μ*m kidney sections were stained with standard periodic acid-Schiff (PAS) protocols to assess renal morphology. The overall magnitude of tubular damage was scored on the basis of the percentage of affected tubules by light microscopy in a high-power field (five fields per animal) by investigators who were unaware of the treatment groups as described previously (Khan et al. 2011).

### Quantification of apoptosis and neutrophil infiltration in the kidney

Apoptosis in response to contrast-induced injury in mice, in the presence or absence of PACAP38, was assessed in paraffin-embedded 4-*μ*m mouse kidney sections with the terminal deoxynucleotidyl transferase dUTP nick end labeling (TUNEL) method using a DeadEnd Fluorometric System (Promega) following the manufacturer's protocol. We detected renal neutrophil infiltration by immunostaining kidney sections with a monoclonal rat anti-mouse neutrophil primary IgG antibody (mAb 7/4; Abcam, Cambridge, MA), a horseradish peroxidase-conjugated rabbit anti-rat IgG (ab6734; Abcam), and a 3,3′-diaminobenzidine substrate kit (Abcam) as described previously (Li et al. 2010). The monoclonal rat anti-mouse neutrophil antibody does not recognize resident tissue macrophages.

### Quantification of mRNA levels in the kidney

Total RNA was isolated from mouse kidney cortex and cleaned up with Qiagen RNeasy® Mini Kits (Valencia, CA) with a DNase treatment step. The quantification was made using a Nanodrop device from Thermo Scientific (Wilmington, DE). For reverse transcriptase-polymerase chain reaction (RT-PCR), 1 *μ*g of RNA was used for cDNA synthesis and real-time 40-cycle PCR was performed with an RT^2^ real-time qPCR SYBR green/ROX kit (SuperArray Biosciences, Valencia, CA) on a Stratagene Mx3005P qPCR System (Santa Clara, CA).

### Quantification of inflammatory cytokine levels in the kidney

The levels of inflammatory cytokines such as monocyte chemotactic protein 1 (MCP-1, CCL2), interferon (IFN)-*γ*, and transforming growth factor (TGF)-*β*1 were measured in mouse kidney cortices from each group using ELISA kits from eBiosciences (San Diego, CA), whereas the levels of tumor necrosis factor-*α* (TNF-*α*) were measured in both serum and kidney cortex.

### Statistical analyses

The results were expressed as the mean ± SEM. Multiple comparisons were analyzed using a one-way analysis of variance (ANOVA), with Tukey–Kramer post hoc tests and Student's *t*-tests (InStat, GraphPad, San Diego, CA).

## Results

### PACAP38 reduces cytotoxicity caused by contrast media in HK-2 cells

**Figure 1:**
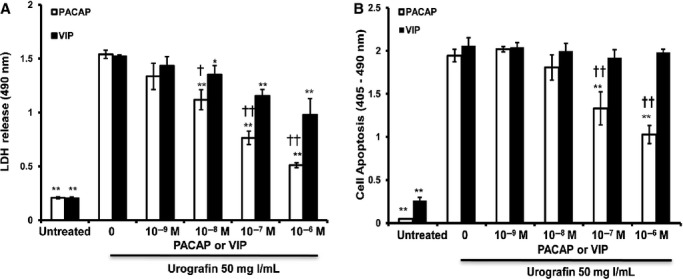
Reduction in contrast-induced renal tubule epithelial cell death by PACAP. (A) Incubation of HK-2 cells with Urografin caused a large increase in the release of LDH into the culture medium. PACAP38 and VIP inhibited the increased release of LDH in a dose-dependent manner. PACAP38 was more potent than VIP. (B) Incubation of HK-2 cells with Urografin caused a very large increase in apoptosis. PACAP38 inhibited the increased apoptosis of HK-2 cells in a dose-dependent manner. *P < 0.05 and **P < 0.01 compared to the cells treated only with Urografin. ^†^*P* < 0.05 and ^††^*P* < 0.01 compared to the cells treated with the same dose of VIP. Bars represent the mean ± SD. PACAP, pituitary adenylate cyclase-activating polypeptide; LDH, lactate dehydrogenase; VIP, vasoactive intestinal peptide; HK-2, human kidney-2.

The effects of both ionic (Urografin) and nonionic (iohexol) contrast media on renal proximal tubule epithelial cells were examined first in vitro. Incubation of confluent HK-2 cells with either contrast medium increased the release of LDH into the culture medium in a dose- and time-dependent manner. Urografin was more cytotoxic than iohexol (data not shown). Incubation of HK-2 cells with 50 mg I/mL of Urografin for 24 h significantly (*P* < 0.01) increased the release of LDH into the culture medium (1.539 ± 0.039 Abs) compared to the untreated cells (0.206 ± 0.008 Abs) (Fig. 1A). Treatment of Urografin-exposed HK-2 cells with either PACAP38 or VIP inhibited the release of LDH into the culture medium in a dose-dependent manner. Both PACAP38 and VIP at a dose range of 10^−8^–10^−6^ mol/L significantly (*P* < 0.05 or <0.01) reduced the LDH release, but PACAP38 was significantly more potent than VIP at each of these dosage points (Fig. 1A).

Incubation of HK-2 cells with 50 mg I/mL of Urografin for 24 h significantly (*P* < 0.01) increased apoptosis (1.944 ± 0.072 Abs) in HK-2 cells compared to the untreated cells (0.048 ± 0.001 Abs) (Fig. 1B). Treatment of Urografin-exposed HK-2 cells with PACAP38 significantly (*P* < 0.01) inhibited HK-2 cell apoptosis in a dose-dependent manner, but VIP did not have a significant inhibitory effect on HK-2 cell apoptosis at any concentration tested (Fig. 1B).

### PACAP38 attenuates the inhibition of HK-2 cell proliferation caused by contrast media

**Figure 2:**
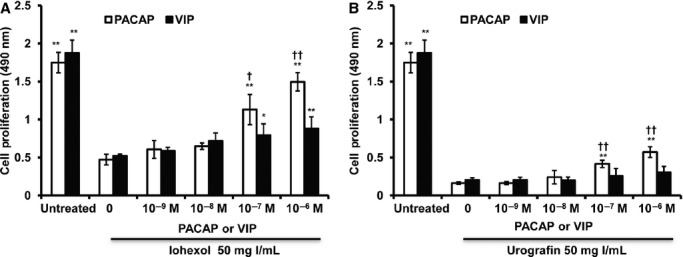
Reduction in contrast-induced inhibition of renal tubule epithelial cell proliferation by PACAP. (A) Incubation of HK-2 cells with iohexol caused a large decrease in the rate of proliferation. PACAP38 and VIP attenuated the decreased in the rate of proliferation in a dose-dependent manner. PACAP38 was more potent than VIP. (B) Incubation of HK-2 cells with Urografin caused a very large decrease in the rate of proliferation. PACAP38 attenuated the decreased in the rate of proliferation in a dose-dependent manner. *P < 0.05 and **P < 0.01 compared to the cells treated only with the same contrast medium. ^†^*P* < 0.05 and ^††^*P* < 0.01 compared to the cells treated with the same dose of VIP. Bars represent the mean ± SD. PACAP, pituitary adenylate cyclase-activating polypeptide; VIP, vasoactive intestinal peptide; HK-2, human kidney-2.

Both iohexol and Urografin at a dose of 50 mg I/mL significantly (*P* < 0.01) inhibited HK-2 cell proliferation compared to the untreated cells, but Urografin appeared to be a much more potent inhibitor of cell proliferation than iohexol (0.184 ± 0.028 vs. 0.497 ± 0.035 Abs) (Fig. 2A and B). Treatment of iohexol-exposed HK-2 cells with either PACAP38 or VIP attenuated the inhibition of HK-2 cell proliferation in a dose-dependent manner. Both PACAP38 and VIP at doses of 10^−7^ and 10^−6^ mol/L significantly (*P* < 0.05 or <0.01) reduced the inhibition of HK-2 cell proliferation, but PACAP38 was significantly more potent than VIP at both dosage points (Fig. 2A). Treatment of Urografin-exposed HK-2 cells with PACAP38 significantly (*P* < 0.01) reduced the inhibition of HK-2 cell proliferation in a dose-dependent manner, but VIP did not have a significant effect on HK-2 cell proliferation at any concentration tested (Fig. 2B).

### PACAP38 reduces release of KIM-1 into HK-2 cell culture medium caused by contrast media

**Figure 3:**
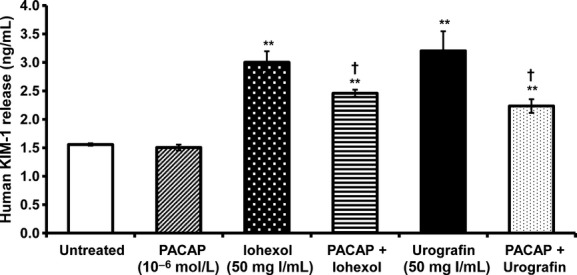
Reduction in contrast-induced renal tubule epithelial cell injury by PACAP. Incubation of HK-2 cells with either iohexol or Urografin caused a large increase in the release of KIM-1 into the culture medium. PACAP reduced the contrast-induced release of KIM-1 into the culture medium. **P < 0.01 compared to the untreated cells. ^†^*P* < 0.05 compared to the cells treated with the same contrast medium. Bars represent the mean ± SD. PACAP, pituitary adenylate cyclase-activating polypeptide; KIM-1, kidney injury molecule 1.

Incubation of HK-2 cells with PACAP38 for 24 h did not have any effect on the release of KIM-1 into the culture medium (Fig. 3). Incubation of HK-2 cells with either iohexol or Urografin for 24 h significantly (*P* < 0.01) increased the release of KIM-1 into the culture medium (3.002 ± 0.195 or 3.203 ± 0.345 ng/mL, respectively) compared to the untreated cells (1.558 ± 0.021 ng/mL). Treatment of either iohexol-exposed or Urografin-exposed HK-2 cells with PACAP38 significantly (*P* < 0.05) reduced the release of KIM-1 into the culture medium (Fig. 3).

### PACAP38 prevents renal insufficiency caused by Urografin

**Figure 4:**
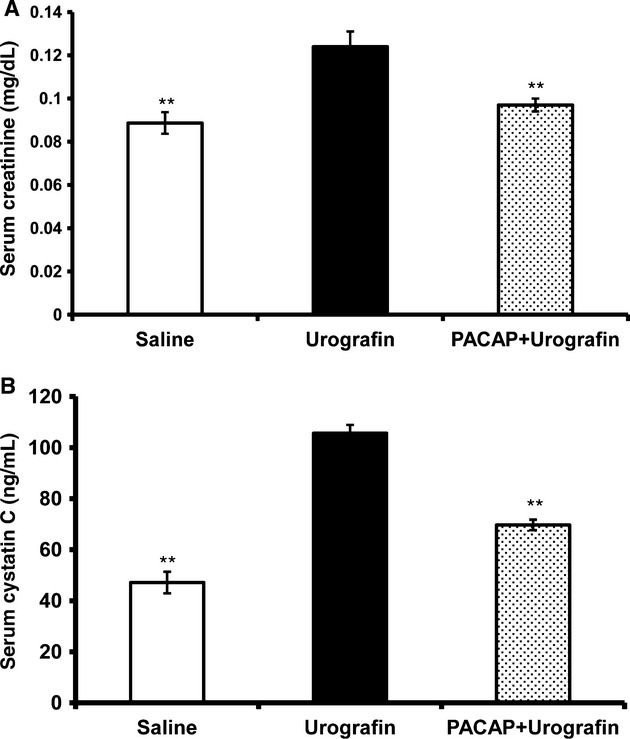
Reduction in contrast-induced impairment of kidney function by PACAP. Treatment of eNOS-deficient mice with Urografin caused an elevation in the serum levels of creatinine (A) and cystatin C (B). PACAP38 reduced the elevated serum levels of creatinine (A) and cystatin C (B) in eNOS-deficient mice caused by treatment with Urografin. **P < 0.01 compared to the mice treated only with Urografin. Bars represent the mean ± SE. PACAP, pituitary adenylate cyclase-activating polypeptide; eNOS, endothelial nitric oxide synthase.

The administration of Urografin to eNOS-deficient mice caused a significant (*P* < 0.01) increase in serum creatinine levels (0.124 ± 0.007 mg/dL) compared to saline-treated eNOS-deficient mice (0.089 ± 0.005 mg/dL). eNOS-deficient mice treated with both Urografin and PACAP38 had serum creatinine levels (0.097 ± 0.003 mg/dL) that were significantly (*P* < 0.01) lower than those of Urografin-treated eNOS-deficient mice and similar to those of saline-treated eNOS-deficient mice (Fig. 4A). The administration of Urografin to eNOS-deficient mice also caused a significant (*P* < 0.01) increase in serum cystatin C levels (105.6 ± 3.26 ng/mL) compared to saline-treated eNOS-deficient mice (47.13 ± 4.21 ng/mL). eNOS-deficient mice treated with both Urografin and PACAP38 had serum cystatin C levels (69.67 ± 2.06 ng/mL) that were significantly (*P* < 0.01) lower than those of Urografin-treated eNOS-deficient mice (Fig. 4B).

### PACAP38 reduces tubular damage, apoptosis, and neutrophil infiltration in the kidney caused by Urografin

**Figure 5:**
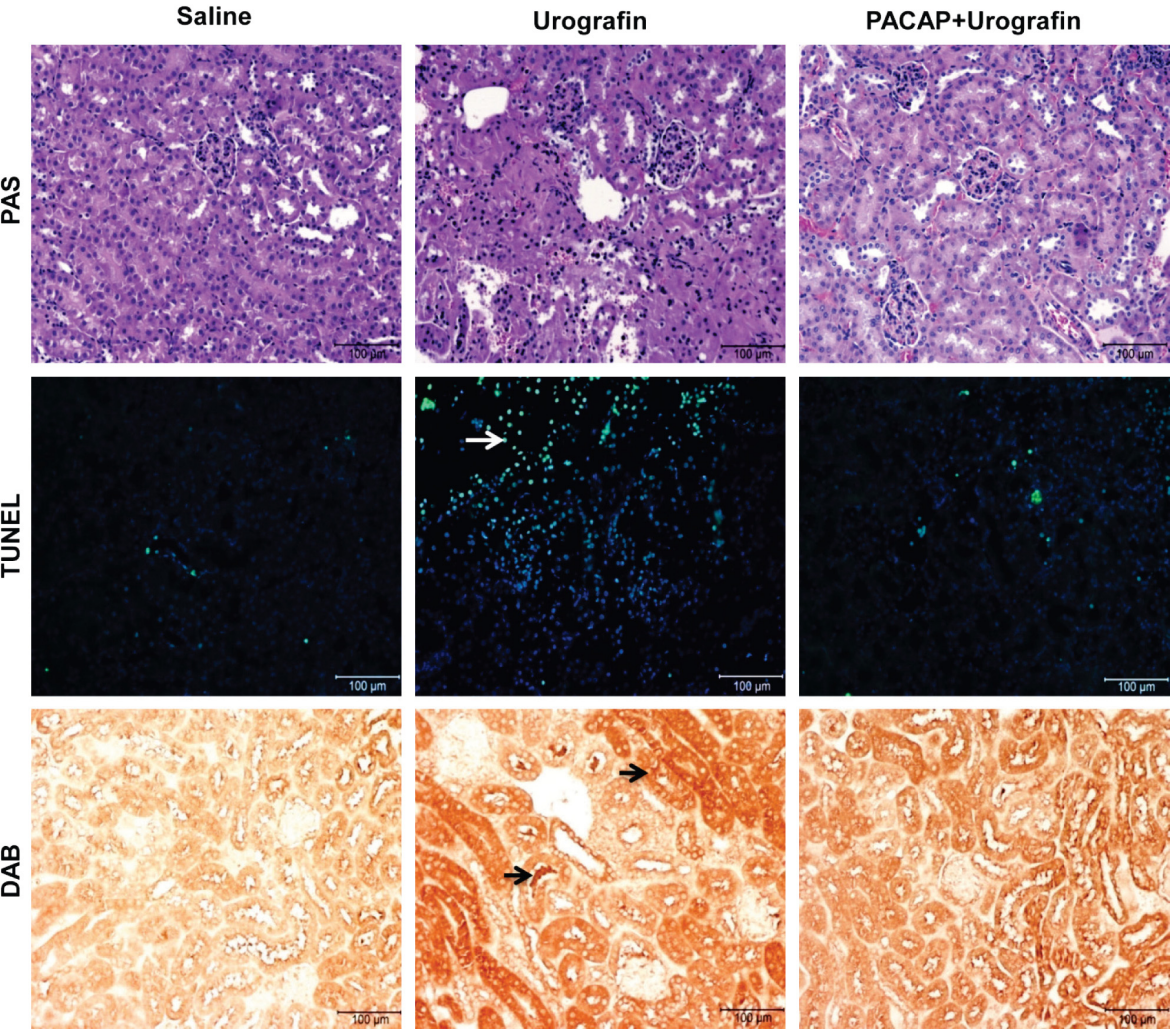
Reduction in contrast-induced morphological kidney injury by PACAP. Representative 4-*μ*m sections of the kidney cortex. Treatment of eNOS-deficient mice with Urografin caused tubular damage (PAS), apoptosis (TUNEL), and neutrophil infiltration (DAB). Treatment with PACAP38 prevented Urografin-induced tubular damage, and reduced apoptosis and neutrophil influx in eNOS-deficient mice. White arrow indicates a fluorescein isothiocyanate-labeled proximal tubule epithelial cell with fragmented chromosomes and black arrows indicate the locations of cells that are immunopositive for the neutrophil marker myeloperoxidase. Scale bars = 100 *μ*m. PAS, periodic acid-Schiff stain; TUNEL, terminal deoxynucleotidyl transferase dUTP nick end labeling stain; DAB, immunohistochemical method with 3,3′-diaminobenzidine as the chromogen; PACAP, pituitary adenylate cyclase-activating polypeptide; eNOS, endothelial nitric oxide synthase.

Periodic acid-Schiff-stained sections of kidneys from Urografin-treated eNOS-deficient mice showed extensive damage to proximal tubules, while similar damage to proximal tubules was not seen in PAS-stained kidney sections from saline-treated eNOS-deficient mice. PAS-stained sections of kidneys from eNOS-deficient mice treated with both Urografin and PACAP38 showed much less damage to proximal tubules than those from Urografin-treated eNOS-deficient mice (Fig. 5). TUNEL-processed sections of kidneys from Urografin-treated eNOS-deficient mice showed a large number of apoptotic (fluorescein isothiocyanate-labeled) cells, whereas apoptotic cells were rarely seen in TUNEL-processed kidney sections from saline-treated eNOS-deficient mice. TUNEL-processed sections of kidneys from eNOS-deficient mice treated with both Urografin and PACAP38 showed far fewer apoptotic cells than those from Urografin-treated eNOS-deficient mice (Fig. 5). Diaminobenzidine (DAB)-stained sections of kidneys from Urografin-treated eNOS-deficient mice showed scattered neutrophil-like cells throughout the kidney cortex, while neutrophil-like cells were rarely seen in the kidney cortex in DAB-stained kidney sections from saline-treated eNOS-deficient mice. DAB-stained sections of kidneys from eNOS-deficient mice treated with both Urografin and PACAP38 showed far fewer neutrophil-like cells than those from Urografin-treated eNOS-deficient mice. Quantitative histological analysis showed significant (*P* < 0.01) Urografin-induced proximal tubule damage (65.00 ± 4.95%) compared to the saline-treated mice (15.9 ± 0.64%). eNOS-deficient mice treated with both Urografin and PACAP38 had significantly (*P* < 0.01) less proximal tubule damage (26.7 ± 1.66%) than Urografin-treated eNOS-deficient mice.

### PACAP38 reduces inflammation in the kidney caused by Urografin

**Figure 6:**
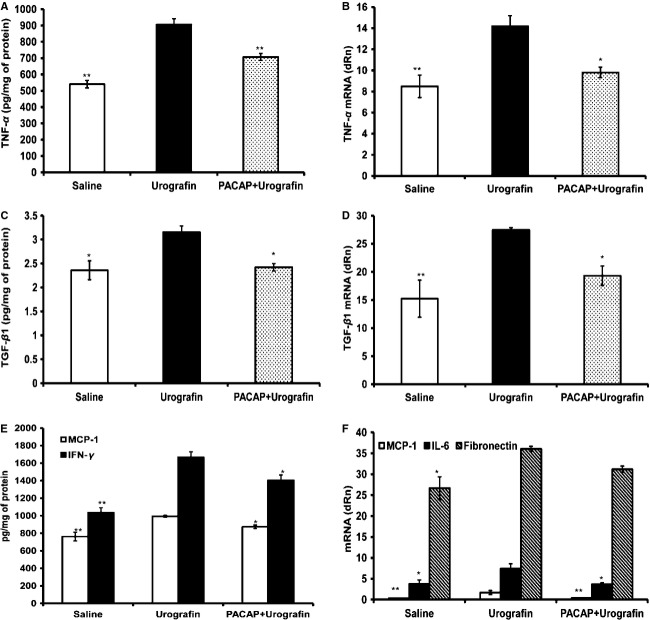
Reduction in contrast-induced inflammation by PACAP. Treatment of eNOS-deficient mice with Urografin caused an increase in the kidney levels of TNF-*α* (A), TGF-*β*1 (C), MCP-1 (E), and IFN-*γ* (E). The kidney levels of the mRNA for TNF-*α* (B), TGF-*β*1 (D), and MCP-1 (F) were also increased in the Urografin-treated mice. Treatment of eNOS-deficient mice with PACAP reduced the Urografin-induced changes in both kidney protein and mRNA levels of these cytokines (A–F). The kidney mRNA levels of Il-6 and fibronectin were also increased by treatment with Urografin (F). *P < 0.05 and **P < 0.01 compared to the mice treated only with Urografin. Bars represent the mean ± SE. PACAP, pituitary adenylate cyclase-activating polypeptide; eNOS, endothelial nitric oxide synthase.

The administration of Urografin to eNOS-deficient mice caused a significant (*P* < 0.05 or <0.01) increase in kidney protein levels of TNF-*α* (905 ± 36.26 vs. 539.951 ± 22.89 pg/mg), TGF-*β*1 (3.153 ± 0.133 vs. 2.359 ± 0.197 pg/mg), IFN-*γ* (1666 ± 62.21 vs. 1036.82 ± 54.58 pg/mg), and MCP-1 (993.5 ± 11.25 vs. 761.604 ± 49.769 pg/mg) compared to saline-treated eNOS-deficient mice (Fig. 6A, C, and E). The kidney levels of the mRNAs for TNF-*α*, TGF-*β*1, MCP-1, interleukin (IL)-6, and fibronectin were significantly (*P* < 0.05 or <0.01) increased in Urografin-treated eNOS-deficient mice compared to saline-treated eNOS-deficient mice (Fig. 6B, D, and F). eNOS-deficient mice treated with both Urografin and PACAP38 had significantly (*P* < 0.05 or <0.01) lower kidney levels of TNF-*α*, TGF- *β*1, IFN-*γ*, and MCP-1 than Urografin-treated eNOS-deficient mice (Fig. 6A, C, and E). The kidney mRNA levels for TNF-*α*, TGF-*β*1, MCP-1, and IL-6 were also significantly (*P* < 0.05 or <0.01) lower in eNOS-deficient mice treated with both Urografin and PACAP38 than in Urografin-treated eNOS-deficient mice (Fig. 6B, D, and F).

### PACAP38 reduces injury, leukocyte infiltration, and apoptosis in the kidney caused by Urografin

**Figure 7:**
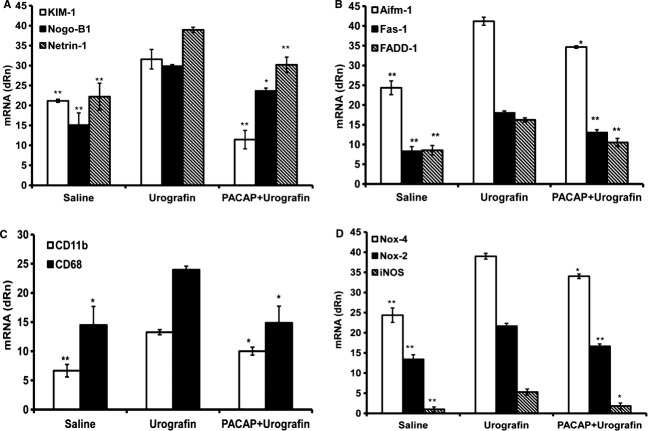
Reduction in contrast-induced kidney injury, leukocyte infiltration, and apoptosis by PACAP. (A) Treatment of eNOS-deficient mice with Urografin caused an increase in the kidney mRNA levels of the kidney injury biomarkers KIM-1, Nogo B1, and netrin 1. Treatment of eNOS-deficient mice with PACAP reduced the Urografin-induced changes in the mRNA levels of all three kidney injury biomarkers. (B) Treatment of eNOS-deficient mice with Urografin caused an increase in the mRNA levels of the proapoptotic genes Aifm 1, Fas 1, and FADD 1. Treatment of eNOS-deficient mice with PACAP reduced the Urografin-induced changes in the mRNA levels of all three proapoptotic genes. (C) Treatment of eNOS-deficient mice with Urografin caused an increase in the kidney mRNA levels for the leukocyte adhesion molecule CD11b and the monocyte/macrophage biomarker CD68. Treatment of eNOS-deficient mice with PACAP reduced the Urografin-induced changes in the mRNA levels of both biomarkers for mononuclear cell infiltration. (D) Treatment of eNOS-deficient mice with Urografin caused an increase in the kidney mRNA levels for enzymes responsible for the synthesis of reactive oxygen and nitrogen species. Treatment of eNOS-deficient mice with PACAP reduced the Urografin-induced changes in the mRNA levels for all three enzymes. *P < 0.05 and **P < 0.01 compared to the mice treated only with Urografin. Bars represent the mean ± SE. PACAP, pituitary adenylate cyclase-activating polypeptide; eNOS, endothelial nitric oxide synthase; KIM-1, kidney injury molecule 1.

The administration of Urografin to eNOS-deficient mice caused a significant (*P* < 0.01) increase in kidney mRNA levels for the kidney injury biomarkers KIM-1, Nogo-B1 (reticulon 4B), and netrin 1 compared to saline-treated eNOS-deficient mice (Fig. 7A). eNOS-deficient mice treated with both Urografin and PACAP38 had significantly lower kidney mRNA levels of KIM-1 (*P* < 0.01), Nogo-B1 (*P* < 0.05) and netrin 1 (*P* < 0.01) than Urografin-treated eNOS-deficient mice. The administration of Urografin to eNOS-deficient mice caused a significant (*P* < 0.01) increase in kidney mRNA levels for the proapoptotic genes Aifm 1, Fas 1, and FADD 1 compared to saline-treated eNOS-deficient mice (Fig. 7B). eNOS-deficient mice treated with both Urografin and PACAP38 had significantly lower kidney mRNA levels for Aifm 1 (*P* < 0.05), Fas 1 (*P* < 0.01), and FADD 1 (*P* < 0.01) than Urografin-treated eNOS-deficient mice. The administration of Urografin to eNOS-deficient mice caused a significant increase in kidney mRNA levels for the leukocyte adhesion molecule CD11b (*P* < 0.01) and the monocyte/macrophage biomarker CD68. (*P* < 0.05) compared to saline-treated eNOS-deficient mice (Fig. 7C). eNOS-deficient mice treated with both Urografin and PACAP38 had significantly (*P* < 0.05) lower kidney mRNA levels for CD11b and CD68 than Urografin-treated eNOS-deficient mice. The administration of Urografin to eNOS-deficient mice caused a significant (*P* < 0.01) increase in kidney mRNA levels for enzymes responsible for the biosynthesis of reactive oxygen and nitrogen species compared to saline-treated eNOS-deficient mice (Fig. 7D). eNOS-deficient mice treated with both Urografin and PACAP38 had significantly lower kidney mRNA levels for NADPH oxidase (Nox)-2 (gp91^phox^, *P* < 0.01), Nox-4 (Renox, *P* < 0.05), and inducible nitric oxide synthase (iNOS) (*P* < 0.05) than Urografin-treated eNOS-deficient mice.

### Molecules and pathways mediating the reduction in Urografin-induced acute kidney injury by PACAP38

**Figure 8:**
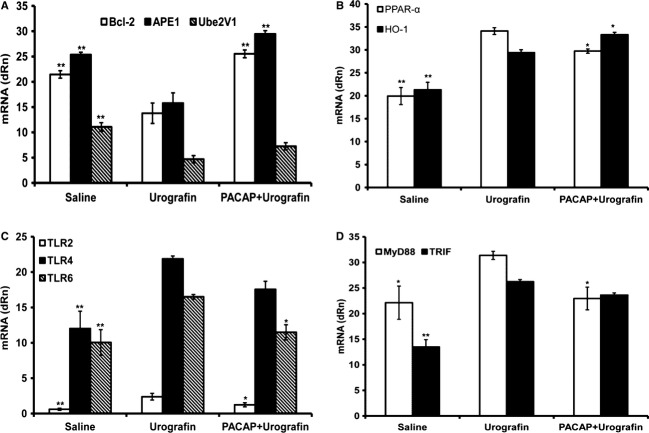
Mechanisms and pathways responsible for reduction in Urografin-induced acute kidney injury. (A) Treatment of eNOS-deficient mice with Urografin caused a decrease in the kidney mRNA levels for the antiapoptotic proteins Bcl-2, APE1, and Ube2V1. Treatment of eNOS-deficient mice with PACAP reduced the Urografin-induced changes in the mRNA levels of all three antiapoptotic proteins, but the increase in the mRNA for Ube2V1 was not statistically significant. (B) Treatment of eNOS-deficient mice with Urografin caused an increase in the kidney mRNA levels of the nuclear receptor PPAR-*α* and the inducible enzyme HO-1. Treatment of eNOS-deficient mice with PACAP reduced the Urografin-induced change in the mRNA levels of PPAR-*α* but further increased the mRNA levels of HO-1. (C) Treatment of eNOS-deficient mice with Urografin caused an increase in the kidney mRNA levels of the innate immune system pattern recognition receptors TLR2, TLR4, and TLR6. Treatment of eNOS-deficient mice with PACAP reduced the Urografin-induced changes in the mRNA levels of all three pattern recognition receptors, but the decrease in the mRNA level for TLR4 was not statistically significant. (D) Treatment of eNOS-deficient mice with Urografin caused an increase in the kidney mRNA levels of the TLR adaptor proteins MyD88 and TRIF. Treatment of eNOS-deficient mice with PACAP reduced the Urografin-induced change in the mRNA levels for MyD88 and TRIF, but the decrease in the mRNA levels for TRIF was not statistically significant. *P < 0.05 and **P < 0.01 compared to the mice treated only with Urografin. Bars represent the mean ± SE. PACAP, pituitary adenylate cyclase-activating polypeptide; eNOS, endothelial nitric oxide synthase; TLR, Toll-like receptor; PPAR, peroxisome proliferator-activated receptor.

The administration of Urografin to eNOS-deficient mice caused a significant (*P* < 0.01) decrease in kidney mRNA levels for the anti-apoptotic proteins Bcl-2, apurinic/apyrimidinic endonuclease 1 (APE1, redox effector factor-1), and ubiquitin-conjugating enzyme E2 variant 1 (Ube2V1) compared to saline-treated eNOS-deficient mice (Fig. 8A). eNOS-deficient mice treated with both Urografin and PACAP38 had significantly (*P* < 0.01) higher kidney mRNA levels for Bcl-2 and APE1 than Urografin-treated eNOS-deficient mice. The administration of Urografin to eNOS-deficient mice caused a significant (*P* < 0.01) increase in kidney mRNA levels for the nuclear receptor peroxisome proliferator-activated receptor (PPAR)-*α* and the inducible enzyme HO-1 compared to saline-treated eNOS-deficient mice (Fig. 8B). eNOS-deficient mice treated with both Urografin and PACAP38 had significantly (*P* < 0.05) lower kidney mRNA levels for PPAR-*α* and significantly (*P* < 0.05) higher kidney mRNA levels for HO-1 than Urografin-treated eNOS-deficient mice. The administration of Urografin to eNOS-deficient mice caused a significant (*P* < 0.01) increase in kidney mRNA levels for the innate immune system pattern recognition receptors Toll-like receptor (TLR)2, TLR4, and TLR6 compared to saline-treated eNOS-deficient mice (Fig. 8C). eNOS-deficient mice treated with both Urografin and PACAP38 had significantly (*P* < 0.05) lower kidney mRNA levels for TLR2 and TLR6 than Urografin-treated eNOS-deficient mice. The administration of Urografin to eNOS-deficient mice caused a significant (*P* < 0.01) increase in kidney mRNA levels for the TLR adaptor proteins MyD88 (*P* < 0.05) and TRIF (*P* < 0.01) compared to saline-treated eNOS-deficient mice (Fig. 8D). eNOS-deficient mice treated with both Urografin and PACAP38 had significantly (*P* < 0.05) lower kidney mRNA levels for MyD88 than Urografin-treated eNOS-deficient mice.

## Discussion

### A novel preclinical model of contrast-induced nephropathy

The development of therapeutic agents for the prevention of contrast-induced nephropathy has been hampered by the lack of a simple and reproducible mouse model. Wild-type mice, like healthy humans, are resistant to contrast-induced nephropathy. The inhibition of nitric oxide synthases and cyclooxygenases with N^G^-nitro-l-arginine methyl ester (l-NAME) and indomethacin, respectively, has been used to make mice and rats more sensitive to iodinated radiocontrast media (e.g., Billings et al. 2008 and Wu et al. 2013). However, these two nonspecific inhibitors interfere with the mechanisms that normally mediate contrast-induced nephropathy and add multiple sources of variance to the experiments. Homozygous eNOS-deficient mice have been reported to be more susceptible to endotoxin-induced acute renal failure than wild-type mice (Wang et al. 2004). We have, therefore, used homozygous eNOS-deficient mice, which have increased blood pressure and decreased heart rate compared to wild-type mice (Shesely et al. 1996), to produce a simple and reproducible mouse model of contrast-induced nephropathy (Figs. 8). The risk factors for contrast-induced nephropathy are additive (McCullough 2008). Therefore, it should be possible to make homozygous eNOS-deficient mice even more sensitive to iodinated contrast agents by adding risk factors. For example, eNOS-deficient mice could be pretreated with streptozotocin or treated with the iodinated contrast agent at a much older age than in the current study. Mice with homozygous deficiency of both eNOS and the leptin receptor might be a more clinically relevant model for the patient population that is at the highest risk for developing contrast-induced nephropathy.

### PACAP has a direct protective effect on renal proximal tubule epithelial cells

Urografin was chosen as the iodinated contrast agent for the in vivo experiments because of its known high toxicity. We have assumed that if PACAP can protect the kidney against a highly toxic iodinated contrast agent such as Urografin, it is likely to protect the kidney against a less toxic iodinated contrast agent such as iohexol.

Pituitary adenylate cyclase-activating polypeptide has both direct and indirect protective effects on renal epithelial and endothelial cells. Pituitary adenylate cyclase-activating polypeptide 38 protected human renal proximal tubule epithelial (HK-2) cells against the toxic effects of both ionic and nonionic iodinated contrast media (Figs. 1–3). PACAP38 also promoted the synthesis of the several cytoprotective proteins in the mouse kidney in vivo (Fig. 8A and B). Previous studies have shown that PACAP and VIP protect HK-2 cells against many other nephrotoxic molecules, including cisplatin (Li et al. 2011), gentamicin (Li et al. 2008), cyclosporine A (Khan et al. 2011), methotrexate (Coy et al. 2011), and hydrogen peroxide (Vacas et al. 2012). Rácz et al. (Rácz et al. 2007) have shown that PACAP also protects endothelial cells against apoptosis caused by hydrogen peroxide (*cf*. Koh and Waschek 2000).

### PACAP has an indirect protective effect on kidney cells

Pituitary adenylate cyclase-activating polypeptide has been shown to inhibit the induction of iNOS in activated macrophages (Delgado et al. 1999b), to inhibit the production of the pro-inflammatory cytokines TNF-*α*, IL-6, and IL-12 in activated macrophages (Ganea and Delgado 2002), and to stimulate the production of the anti-inflammatory cytokines IL-4 and IL-10 in activated macrophages (Ganea and Delgado 2002). PACAP also inhibited the production of TGF-*β*1 in activated macrophages (Sun et al. 2000). PACAP inhibited the proliferation of B-cell (Arimura et al. 2006) and T-cell (Coy et al. 2011) lines, and inhibited the phytohemagglutinin-stimulated secretion of IL-2 from T cells (Coy et al. 2011). These in vitro experiments show that PACAP has a direct suppressive effect on cells of the immune system and, therefore, should indirectly protect epithelial and endothelial cells in the kidney against immune system-mediated injury.

Consistent with the above in vitro experiments, PACAP38 reduced the Urografin-induced innate immune responses in the mouse kidney in vivo (Figs. 6, 7C, and 8C). PACAP38 also reduced the Urografin-induced increase in the mRNA levels in the kidney for enzymes responsible for the synthesis of reactive oxygen and nitrogen species (Fig. 7D). The suppression of the Urografin-induced innate immune responses in the kidney was associate with a reduction in the Urografin-induced increase in biomarkers for kidney epithelial and endothelial cell injury (Fig. 7A) and apoptosis (Fig. 7B). These results also show that the innate immune system plays a major role in the pathogenesis of contrast-induced nephropathy.

### PACAP affects renal blood flow and ischemic kidney damage

Both ionic and nonionic contrast media reduce blood flow in the kidney. Hypoperfusion of the kidney, especially the renal medulla, is a major cause of contrast-induced kidney dysfunction (Heyman et al. 2008; Sendeski 2011). PACAP has been reported to increase blood flow in the kidney (Nilsson 1994). In addition, PACAP inhibits platelet aggregation via the VPAC_1_ receptor (Freson et al. 2004). Therefore, PACAP should attenuate the hypoperfusion of the kidney, especially the renal medulla, caused by iodinated contrast media. In addition, PACAP has been shown to reduce the kidney damage caused by I/R (Khan et al. 2012). The current experiments and the previous literature indicate that PACAP should counteract multiple processes that contribute to the pathogenesis of contrast-induced nephropathy, that is, PACAP should reduce the direct toxic effects of iodinated contrast media on renal epithelial and endothelial cells, suppress the innate immune responses in the kidney to iodinated contrast media, and attenuate the decrease in renal blood flow caused by iodinated contrast media. In addition, PACAP would be expected to decrease the severity of any anaphylactic reaction caused by iodinated contrast media (Delgado et al. 1999a).

### Kidney injury biomarkers

Ichimura et al. (Ichimura et al. 1998) reported that KIM-1 mRNA and protein were dramatically upregulated in renal proximal tubule epithelial cells following I/R. We found a large increase in KIM-1 in the culture medium of HK-2 cells incubated with either ionic or noninonic contrast media (Fig. 3) and a large increase in KIM-1 mRNA in the kidneys of mice treated with Urografin (Fig. 7A). Sohn et al. (Sohn et al. 2013) found similar large increases of KIM-1 in the culture medium of HK-2 cells incubated with cisplatin and KIM-1 mRNA in the kidneys of rats treated with cisplatin. Sohn et al. (Sohn et al. 2013) also reported a significant increase of KIM-1 in the urine of cisplatin-treated rats. Marin et al. (Marin et al. 2010) reported that Nogo-B mRNA and protein were dramatically upregulated in renal proximal tubule epithelial cells following unilateral ureteral obstruction. We found a similar large increase in Nogo-B mRNA in the kidneys of mice treated with Urografin (Fig. 7A). Mice treated with both PACAP38 and Urografin had significantly lower levels of KIM-1 and Nogo-B mRNA than mice treated only with Urografin (Fig. 7A). Whether the concentration of Nogo-B in serum or urine is increased following acute kidney injury is unknown.

### PACAP is a potential therapeutic agent for contrast-induced nephropathy

Contrast-induced nephropathy is an independent predictor of increased short-term and long-term mortality (e.g., From et al. 2008). The number of patients treated with iodinated contrast media is expected to increase in the coming years because of both the aging of the general population and the more frequent use of contrast-enhanced imaging techniques. The antioxidants N-acetylcysteine, vitamin E (*α*-tocopherol), and ascorbic acid have been used as adjunctive cytoprotective agents with iodinated contrast media in large or small clinical trials without any consistent clear cut evidence of benefit (McCullough 2008; McCullough et al. 2011). Vasodilators and vasoconstrictor antagonists, including fenoldopam, SB 209670, atrial natriuretic peptide, theophylline, and aminophylline, have also been used as adjunctive cytoprotective agents with iodinated contrast media in small clinical trials without any consistent clear cut evidence of benefit (McCullough 2008; Thomsen et al. 2008). The intravenous administration of isotonic fluids is the only generally accepted strategy to reduce the incidence of contrast-induced nephropathy (Mueller et al. 2002; Thomsen et al. 2008). There are no drugs that have been approved by the Food and Drug Administration (FDA) specifically for the prevention of contrast-induced nephropathy.

Pituitary adenylate cyclase-activating polypeptide has already been administered to healthy human volunteers by investigators in at least five different laboratories (Warren et al. 1992; Hammond et al. 1993; Chiodera et al. 1996; Filipsson et al. 1997; Doberer et al. 2007) and to a patient with multiple myeloma under a U.S. FDA-approved protocol (Li et al. 2007) without any indication of serious side effects. These clinical studies and the current preclinical experiments strongly suggest that PACAP would be both safe and effective for the prevention of contrast-induced nephropathy.

## Conclusions

Homozygous eNOS-deficient mice are a simple and reproducible preclinical model for studying contrast-induced nephropathy. PACAP protects renal proximal tubule epithelial cells in vitro against the toxicity caused by ionic and nonionic contrast media. PACAP protects the structure and function of the kidney in vivo against the deleterious effects of the ionic contrast medium Urografin. PACAP is a potential therapeutic agent for contrast-induced nephropathy.
